# Barriers and facilitators to bracing in adults with painful degenerative scoliosis: a single-centred mixed-method feasibility study

**DOI:** 10.1186/s12891-022-06111-0

**Published:** 2023-01-16

**Authors:** Flora Dureigne, Marie-Ombeline Chagnas, Alexandra Roren, Emmanuel Couzi, Marie-Martine Lefèvre-Colau, Sylvain Moreau, Vanina Nicol, François Rannou, Camille Daste, Christelle Nguyen

**Affiliations:** 1 AP-HP.Centre-Université Paris Cité, Rééducation et Réadaptation de l Appareil Locomoteur et des Pathologies du Rachis, 75014 Paris, France; 2 INSERM UMR 1153, Centre de Recherche Épidémiologie et Statistique Paris Sorbonne, ECaMO Team, 75004 Paris, France; 3 Fédération pour la Recherche sur le Handicap et l Autonomie, 75013 Paris, France; 4 Université Paris Cité, Faculté de Santé, UFR de Médecine, 75006 Paris, France; 5 INSERM UMR-S 1124, Toxicité Environnementale, Cibles Thérapeutiques, Signalisation Cellulaire (T3S), Campus Saint-Germain-des-Prés, 75006 Paris, France; 6 INSERM UMR 1153, Centre de Recherche Épidémiologie et Statistique Paris Sorbonne, METHODS Team, 75004 Paris, France

## Abstract

**Background:**

Conservative treatments including bracing and exercise therapy are prescribed on the first-line in adults with degenerative scoliosis. However, adherence to conservative treatments is low. We aimed to assess barriers and facilitators to bracing in adults with painful degenerative scoliosis.

**Methods:**

We conducted a single-centred mixed-method pilot and feasibility study. All patients scheduled for a multidisciplinary custom-made bracing consultation, from July 2019 to January 2020, in a French tertiary care centre, were screened. Patients were eligible if they had painful adult degenerative scoliosis and a prescription for a rigid custom-made lumbar-sacral orthosis. The primary outcome was barriers and facilitators to bracing assessed by a qualitative approach using semi-structured interviews. Secondary outcomes were back pain, spine-specific activity limitations, symptoms of depression and satisfaction with bracing post-intervention assessed by a quantitative approach.

**Results:**

Overall, 56 patients were screened and 14 (25%) were included. Mean age was 68.2 (12.3) years. Mean follow-up was 9.8 (2.0) months. Barriers to bracing were increased limitations in some activities, discomfort in hot weather and burden of aesthetic appearance. Facilitators to bracing were reduced pain, improved activities of daily living, suitable weight and improved spinal alignment. Participants self-implemented solutions to enhance adherence. The mean reduction from baseline in pain intensity was 1.7 (2.3) of 10 points, and 6 of 13 patients (46%) had pain intensity < 4 of 10 points.

**Conclusion:**

Bracing is a feasible intervention for people with painful adult degenerative scoliosis. Patients self-implemented their own solutions to enhance adherence.

**Supplementary Information:**

The online version contains supplementary material available at 10.1186/s12891-022-06111-0.

## Introduction

Adult degenerative scoliosis is a common spinal condition. It is a three-dimensional deformity of the spine, that associates malalignment of the spine in the coronal and sagittal planes and vertebral rotation in the axial plane [1, 2]. The mean age at presentation is 70 years. The prevalence of adult degenerative scoliosis is between 32% [3] and 68% [4]. Its burden is increasing in ageing population [5]. Monitoring of adult degenerative scoliosis relies on clinical and radiological examinations [6], and is recommended every one to five years [7]. There are two types of adult degenerative scoliosis: progressive adolescent idiopathic scoliosis [5] and de novo scoliosis. This latter develops after skeletal maturity and results from degenerative spinal changes [7]. Individuals can remain asymptomatic for several years [4]. The main symptoms are back pain, sometimes associated with radiculalgia, and activity limitations and participation restriction [7, 8].

The first-line treatment of adult degenerative scoliosis is conservative. It includes analgesics, bracing and exercise therapy [9]. Bracing is prescribed to alleviate pain but also supposedly to improve spinal alignment and/or slow down structural progression of the spinal deformity. In a retrospective study of 38 patients with adult scoliosis and a mean follow-up time of 22 years, Palazzo and colleagues reported that bracing may be effective in slowing down the rate of progression in adult scoliosis [5] and suggested a minimum number of hours of daily wear of 6 hours. However, adherence to bracing commonly decreases over time [10], and only 24% of the general practitioners feel comfortable to follow its prescription [11]. Further, guidelines for bracing in adults, based on high level of evidence do not exist. Therefore, a deeper understanding of barriers and facilitators to bracing is needed.

In the present study, we aimed to assess some aspects of the feasibility of bracing in adults with painful degenerative scoliosis, namely barriers and facilitators.

## Methods

### Design

We conducted a mixed-method pilot and feasibility study in a single French tertiary care centre (Departement of Physical and Rehabilitation Medicine, Cochin Hospital, Paris), that falls under the conceptual framework of feasibility as defined by Eldridge and colleagues [12]. Our study is reported in accordance with the STROBE checklist **(**Appendix 1) and COREQ guidelines [13, 14] (Appendix 2). Our internet survey is reported in accordance with the CHERRIES checklist (Appendix 3). All eligible patients who had a wearing brace referral between July 2019 and January 2020 were interviewed between July and September 2020. The mean time elapsed between referral and a follow-up interview for assessments was 9.8 (2.0) months.

### Participants

Patients who had a multidisciplinary custom-made bracing consultation from July 2019 to January 2020 were consecutively screened. Those who fulfilled inclusion criteria were invited to participate in the study. Inclusion criteria were: 1/ age ≥ 40 years old, 2/ painful adult degenerative scoliosis (idiopathic or de novo), and 3/ having had a prescription for a rigid custom-made lumbar-sacral orthosis. Exclusion criteria were: 1/ spinal deformity secondary to a specific cause, 2/ insufficient proficiency in French, 3/ cognitive disorders, 4/ refusal to participate in the study, and 5/ patients under guardianship or curatorship. Individuals who were already using a brace were eligible, so as those for whom it was a new and first-time wearing brace referral.

### Intervention

Multidisciplinary custom-made bracing consultation is designed to discuss prescription of custom-made brace in adults with spinal disorders and to implement measures to enhance adherence, as appropriate. It involves senior physicians in physical and rehabilitation medicine (EC,VN, CD, CN) with experience in prescribing custom-made bracing (prescription of ≥5 custom-made braces a month) and senior orthotists (SM, SG), in presence of the patients and their families. Firstly, indication of custom-made bracing is considered and discussed between health care providers and patients based on the assessments of impairments and activities and participation and the risk of progression of the deformity, as well as the willingness of patients to be treated with bracing. Secondly, if indication to bracing is retained, specific oral and written advice and follow-up are offered at the time of the brace prescription to enhance adherence to bracing and to monitor efficacy and safety. Patients are also instructed to contact the orthotist and/or the physicians as needed to adapt the brace.

### Qualitative assessments

A provisional questionnaire was elaborated by a senior general practitioner (MOC) and a senior physician in physical and rehabilitation medicine (CN) in order to collect barriers and facilitators to bracing, as experienced by patients. The provisional questionnaire was reviewed by senior physicians (EC, MMLC, VN, FR, CD), physiotherapists (AR) and orthotists (SM, SG). After consensus, five categories were included in the final version of the questionnaire (Appendix 4): 1/ material fabrication, 2/ acceptability of bracing, 3/ side effects and self-reported solutions, 4/ barriers to bracing and self-reported solutions, and 5/ facilitators to bracing.

A single investigator, a female general practitioner (MOC), contacted consecutively all eligible patients by phone from July to September 2020 to invite them to participate in the study. If a patient did not respond after two phone calls on two different days or refused to participate, he was considered as having declined participation. At the beginning of each phone contact, the investigator introduced herself and explained the purpose of the study, then started the interview following the prespecified interview guide (Appendix 4). The mean duration for each interview was 45 min. Each interview was audiorecorded then transcribed. When a participant was not able to answer an open question, answering a checklist was offered. Verbatims were analyzed for thematic analysis by two female investigators (MOC, FD), who received a specific training in qualitative research before the study. The two investigators manually and independently extracted key themes from the verbatims [13, 14]. The individual interview guide was not refined during the study.

### Quantitative assessments

On the same day as the phone contact, the investigator sent an email with a link to the online self-administered questionnaires, as well as an anonymization number. If the self-administered questionnaires were not completed within 2 weeks, a reminding e-mail was sent to patients. If a participant could not complete the self-administered questionnaires online, he/she could complete it by phone with the investigator. If a participant did not complete the questionnaires online or by phone, after at least one reminder, he/she was considered as non-respondent to the quantitative assessments. Five outcomes were assessed post-intervention: 1/ lumbar pain using a numeric rating scale (0, no pain to 10, maximal pain), 2/ radicular pain scale using a numeric rating scale (0, no pain to 10, maximal pain) [15], 3/ spine-specific activity limitations using the Oswestry disability index (ODI, 10 items, each one rated from 0, no limitations, to 5, maximal limitations; a minimal disability corresponds to a score between 0 and 20, a moderate disability between 21 and 40 and a severe disability between 41 and 60) [16, 17]; 4/ symptoms of depression using the patient health questionnaire-2 (PHQ-2, 2 questions, each one rated from 0, no symptoms to 3, symptoms almost every day; if the score is ≥3, major depressive disorder is likely) [18, 19], and 5/ satisfaction with bracing using the Quebec user evaluation of satisfaction with assistive technology (QUEST) questionnaire (12 questions, each one rated from 0, not satisfied at all to 5, very satisfied) [20]. The QUEST questionnaire evaluates the satisfaction of the patient with its assistive technology. We chose this questionnaire because evidence suggests good psychometric properties [21]. Minimum clinically important difference for low back pain intensity, leg pain intensity and Oswestry Disability Index has been previously reported [22], but is not available for PHQ-2 and QUEST questionnaires, yet.

### Statistical analysis

Discrete variables were expressed as absolute frequencies (n/N [%]). Continuous variables were expressed as mean (SD). All analyses were performed using the Excel software.

### Ethical consideration and funding statement

Our study was not funded. It was approved by our institutional review board (CERAPHP, IRB registration #00011928). All participants were informed and gave consent to participate.

## Results

### Participants

**Fig. 1:**
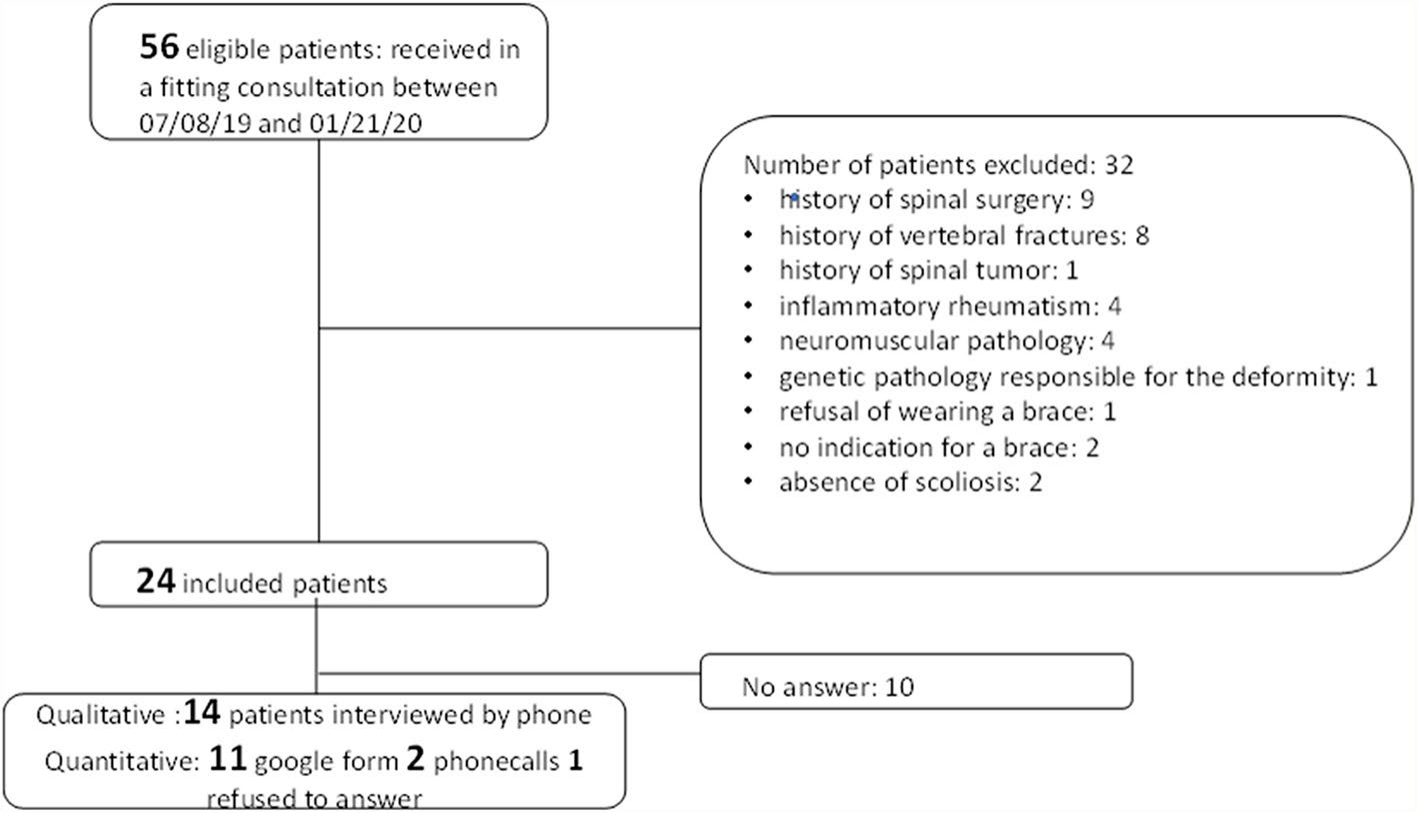
Flow chart

Overall, 56 patients had a multidisciplinary custom-made bracing consultation between July 2019 and January 2020: 24/56 (43%) patients were eligible and contacted by phone, and 14/24 (58%) patients accepted to participate in the study. In total, 14/14 (100%) participants completed qualitative assessments and 13/14 (79%) completed quantitative assessments (Fig. 1). Mean age was 68.2 (12.3) years, mean lumbar pain was 5.3 (2.0) of 10 points, 11/14 (79%) participants were women, 6/14 (43%) had idiopathic scoliosis and 8/14 (57%) had de novo scoliosis (Table 1). The mean time elapsed between the prescription of bracing and qualitative and quantitative assessments was 9.8 (2.0) months. Therefore, there were no newly prescribed brace users.

**Table 1:** Demographics and clinical characteristics of patients with scoliosis (*n* = 14)

Women n (%)	11 (79)
Age (years), mean (SD)	68.2 (12.3)
Body mass index (kg/m^2^), mean (SD)^a^	21.7 (1.7)
Level of education n (%)	
• Unknown	7 (50)
• ≥ High school	6 (43)
• < High school	1 (7)
Professional status, n (%)	
• Retired	9 (64)
• Full time active	4 (29)
• Active part time	1 (7)
Type of scoliosis n (%)	
• De novo	8 (57)
• Progressive adolescent idiopathic scoliosis	6 (43)
Location of scoliosis n (%)	
• Thoraco-lumbar	7 (50)
• Lumbar	5 (36)
• Thoracic	1 (7)
• Double major	1 (7)
Previous spinal injections, n (%)^b^	11 (85)
• Posterior joints	8 (62)
• Epidural	5 (38)
• Intradiscal	1 (8)
• Foraminal	0
Previous non-pharmacological treatments, n (%)	
• Physical therapy	13 (93)
• Home-based exercises	5 (42)
• Balneotherapy^b^	2 (15)
• Temporary plaster cast^c^	6 (43)
Type of rigid brace prescribed, n (%)	
• Thermoformed plastic brace	12/14 (86)
• Plaster cast brace	2/14 (14)
Mean time of wearing brace a day (hours), mean (SD)	4.2 (1.9)
Lumbar pain (0–10), mean (SD)	5.3 (2)
Radicular pain (0–10), mean (SD)	1.4 (2.7)
Height loss (cm), mean (SD)^e^	5.8 (2.9)
Cobb angle (degrees), mean (SD)	25.4 (11.3)
Time elapsed between the prescription of bracing and assessments (months), mean (SD)	9.8 (2.0)

^a^3 missing data

^b^1 missing data

^c^2 missing data

^d^5 missing data

^e^4 missing data

### Primary outcome

Regarding barriers to bracing, bracing could increase limitations in some activities of daily living (“to drive, to sit down”, “to bend over”), was difficult to wear during hot weather (“suffocating with it in summer”) and induced a burden of aesthetic appearance (“difficult to dress with”; “I never wear it outside”) (Table 2). Regarding facilitators to bracing, 12/14 (86%) participants found that bracing relieved pain (“pain less present”; “it relieves me when I stay up”), 11/14 (78%) reported an improvement in activities of daily living (“I can do my activities again [ …] sitting, or cooking, ironing, sewing, gardening”), 10/14 (71%) were satisfied with the weight (“very light”), 9/14 (64%) reported a straightening of the spine and a horizontality of the gaze (“I stand straighter”; “it prevents me from leaning forward”). Participants also found that the maintenance of the brace was easy (“I wash it with a sponge”; “I wash it with water and soap”) (Table 3). Participants self-implemented solutions to overcome most barriers. For example, they adjusted their movements, took a change of clothes in the summer or chose loose fitting clothing (Table 2).

**Table 2:** Primary outcome: barriers to bracing and self-implemented solutions (n = 14)

Barriers	n (%)	Corresponding items	Solutions
Limitation in activities of daily living	8 (57)	“To drive; to sit down” “Like a penguin on the ice floe” “Not able to bend down” “Can’t use it to do sport” “To bend over”	“Adaptation of movements” “The ortho-prosthetist readjusts”
Difficult to wear it during hot weather, excessive sweating	8 (57)	“Suffocating with it in summer” “Impossible to wear it” “I didn’t wear it”	“To take a change of clothes” “Seamless t-shirt” “Cotton tank top”
Burden of aesthetic appearance	7 (50)	“It has an impact on self-image, it has an impact on femininity” “I never wear it outside” “It’s ugly” “The way people look at you” “Difficult to dress with” “Not very stylish”	“Loose-fitting clothing” “I put it on last over my clothes or under my pants”
Digestive discomfort	3 (21)	“I have aerophagia” “I have reflux”	“Loosening the brace” “I do not wear it during meals”
Increased pain	3 (21)	“Muscular pains” “Hips and ribs [pain]”	“Essential oils, analgesics, heat, rest” “I saw the ortho-prosthetist”
Dissatisfaction with the fit	2 (14)	“The brace is much too wide now” “The straps are not long enough”	“I have hernia but the brace had been made according to” “Was modified and it is better since”
Skin discomfort	2 (14)	“I am very careful”	“Cotton t-shirt below under”
Respiratory discomfort	2 (14)	“Yes [respiratory discomfort] but also related to aerophagia”	“Loosening the brace” “I learned to breathe with it”
Asthenia	1 (7)	“Significant fatigue with the brace”	
Urinary discomfort	1 (7)	“Urine loss”	
Communication problems with health care provider	1 (7)	“Have you ever worn a brace? Do you know what you are talking about?”	
Traveling	1 (7)	“[The brace is] cumbersome”

**Table 3:** Primary outcome: facilitators to bracing (n = 14)

Item mentioned	n (%)	Examples
Reduced pain	12 (86)	“Pain less present” “Less painful” “It relieves me when I stay up” « I was able to stop taking anti-inflammatory drugs”
Improved activities of daily living	11 (78)	“I need to put it on to walk” “I can do my activities again […] sitting, or cooking, ironing, sewing, gardening. But also walking, cycling. It is my best friend!”
Suitable weight	10 (71)	“Perfectly acceptable” “More pleasant to wear” “Very light”
Straightening of the spine and horizontality of the gaze	9 (64)	“I stand straighter” “It prevents me from leaning forward” “It straightens me”
Easy to put on and remove	9 (64)	“At first, yes, I had difficulty putting it on”P14
Benefit in daily life	9 (64)	“Increase in my overall comfort” P 8 “I have a feeling of freedom since I have it” P10 “Patients who are reluctant to wear a brace, I show them that I wear one too” P13
Easy to clean	7 (50)	“Sponge” “Tweezers” “Water with soap”
Improvement in walking	6 (43)	“It increased my walking time” P6 “It is easier to walk, more comfortable” P4
Improvement of asthenia	5 (36)	“I am no longer tired” “less tired from my deformity”
Helpful to carry heavy loads	2 (14)	“I can now carry heavy loads.”

### Secondary outcomes

Mean lumbar pain decreased from 5.3 (2.0) of 10 points to 3.7 (2.1) of 10 points (mean reduction of 1.6 [2.3] of 10 points). Mean radicular pain increased from 1.4 (2.7) of 10 points to 2.7 (2.2) of 10 points (mean increase of 1.2 [2.8] of 10 points). At the time of assessments, the mean ODI score was 33.5 (15.6) of 100 points **(**Table 4), 5/13 (38%) participants reported minimal disability and 5/13 (38%) reported severe disability (Appendix 5). The mean PHQ-2 score was 2.2 (0.2) (19) and 5/13 (38%) participants had a PHQ-2 score ≥ 3 (Appendix 6). The mean QUEST score was 4.2 (0.5) and 13/13 (100%) participants were very satisfied or quite satisfied with bracing (Appendix 7).

**Table 4:** Secondary outcomes (n = 13)

	At baseline	At follow-up	Variation
Lumbar pain (0–10), mean (SD)^a^	5.3 (2)	3.7 (2.1)	1.6 (2.3)
Radicular pain (0–10), mean (SD)^a^	1.4 (2.7)	2.7 (2.2)	1.2 (2.8)
ODI (0–100), mean (SD)	Not applicable	33.5 (15.6)	Not applicable
PHQ-2 (0–6), mean (SD)	Not applicable	2.2 (2)	Not applicable
QUEST (0–60), mean (SD)	Not applicable	4.2 (0.5)	Not applicable

*ODI* Oswestry disability index; *PHQ-2* patient health questionnaire-2; *QUEST* Quebec user evaluation of satisfaction with assistive technology;

^a^5 missing data

## Discussion

In the present study, we found that barriers to bracing were increased limitations in some activities, discomfort in hot weather and burden of aesthetic appearance. Facilitators to bracing were reduced pain, improved activities of daily living, suitable weight and improved spinal alignment. Participants self-implemented solutions to enhance adherence. The mean reduction from baseline in pain intensity was 1.7 (2.3) of 10 points at 9.8 (2.0) months.

Efficacy on pain seems to be an important facilitator to bracing. In our study, 12/14 (86%) participants reported being “less painful” with bracing. In a study of 20 individuals treated with bracing, Zaina and colleagues reported a reduction in back pain at one month in 13/20 (65%) participants [23]. Weiss and colleagues also reported a reduction in back pain at 18 months in 56 adults with scoliosis treated with bracing [24].

Interestingly, wearing a brace during activities of daily living was considered both as facilitator (11/14 [78%] participants) but also as a barrier (8/14 [57%] participants). Participants reported improvement in activities requiring prolonged standing (cleaning, cooking, walking) but discomfort in bending forward (putting on shoes, golfing) or prolonged sitting (driving). Using the brace questionnaire, Piantoni and colleagues found in a survey of 43 female participants with idiopathic scoliosis treated with bracing, that 58% had back pain when sitting and 47% when walking. Overall, 46% of participants felt their quality of life deteriorated [25]. During the multidisciplinary custom-made bracing consultation, participants were also advised to adapt their movements when wearing the brace which may have decreased its burden.

The burden of aesthetic appearance was frequent (7/14 [50%] of participants). Piantoni and colleagues reported that 75% of patients had to wear different clothes due to bracing [25]. Consistently, Wang and colleagues reported that self-image was a major determinant of health-related quality of life in adolescents with scoliosis wearing a brace [26]. Involving patients in the design process may enhance their adherence [27].

Even though our study was not primarily designed to assess secondary outcomes, we found positive results regarding lumbar pain, activity limitations, satisfaction with the brace, and symptoms of depression. We also found that adherence to bracing was quite high, with regards to previous reports [5, 10], reaching 60% at 9.8 (2.0) months. All participants agreed to have a prescription of bracing at the multidisciplinary custom-made bracing consultation, which seems important for further adherence and satisfaction with bracing. Multidisciplinary bracing consultation and personalized close follow-up appear also as key determinants of long term adherence.

Our study has limitations. Our sample size was small and one could not analyze whether barriers and facilitators differed with the type of rigid brace. Our study was single-centred and all participants had a multidisciplinary custom-made bracing consultation with experienced physicians and orthotists which may have positively influenced their views about bracing and their adherence. We used open-ended questions, but some participants had difficulty to answer. Using a checklist may have influenced their answers. We did not follow-up evolution of the curves on X-ray which could have been associated with secondary outcomes. However, as prespecified in our protocol, our research was designed only to assess the barriers and facilitators to bracing. Therefore, our study was underpowered to address the progression of the curves. In addition, oour follow-up after brace prescription was short (approximately 10 months). Only a randomized controlled trial appropriately dimensioned, with a longer follow-up, can address a progression criterion, with proper methods and samples. We cannot exclude that body mass index, activity level, and type of scoliosis could play a role in the outcomes assessed. Because of our sample size was small and our design was mixed, our quantitative analyses were limited to the description of changes and could not allow precise analyses of their clinical significance. Finally, our study was conducted during the summer of 2020 heatwave. This specific context may have affected outcomes.

In conclusions, bracing is a feasible intervention for people with painful adult degenerative scoliosis. Patients self-implemented their own solutions to enhance adherence. Based on our dataset, we cannot say our intervention offers promise of an effect. Our findings will be useful to design a large-scaled randomized controlled trial to assess the efficacy of bracing in adults with painful degenerative scoliosis.

## Supplementary Information

**Additional file 1: Appendix 1.** Strengthening the Reporting of OBservational studies in Epidemiology (STROBE) statement. **Appendix 2.** Consolidated criteria for reporting qualitative research (COREQ) checklist. **Appendix 3.** Checklist for Reporting Results of Internet E-Surveys (CHERRIES). **Appendix 4.** Interview guide. **Appendix 5.** Disability according to Oswestry disability index (*n* = 13).

## Data Availability

The data that support the findings of this study are available from Prof. Christelle Nguyen but restrictions apply to the availability of these data, which were used under license for the current study, and so are not publicly available. Data are however available from the authors upon reasonable request and with permission of Prof. Christelle Nguyen (christelle.nguyen2@aphp.fr).

## Abbreviations

SD: standard deviation.

## References

[1] Lou E, Raso JV, Hill DL, Mahood JK, Moreau MJ (2004). Correlation between quantity and quality of orthosis wear and treatment outcomes in adolescent idiopathic scoliosis. Prosthetics Orthot Int.

[2] Millner PA, Dickson RA (1996). Idiopathic scoliosis: biomechanics and biology. Eur Spine J.

[3] Robin GC, Span Y, Steinberg R, Makin M, Menczel J (1982). Scoliosis in the elderly: a follow-up study. Spine (Phila Pa 1976).

[4] Schwab F, Dubey A, Gamez L, El Fegoun AB, Hwang K, Pagala M, Farcy JP (2005). Adult scoliosis: prevalence, SF-36, and nutritional parameters in an elderly volunteer population. Spine (Phila Pa 1976).

[5] Palazzo C, Montigny JP, Barbot F, Bussel B, Vaugier I, Fort D, Courtois I, Marty-Poumarat C (2017). Effects of bracing in adult with scoliosis: a retrospective study. Arch Phys Med Rehabil.

[6] Ailon T, Smith JS, Shaffrey CI, Lenke LG, Brodke D, Harrop JS, Fehlings M, Ames CP (2015). Degenerative spinal deformity. Neurosurgery.

[7] Diebo BG, Shah NV, Boachie-Adjei O, Zhu F, Rothenfluh DA, Paulino CB, Schwab FJ, Lafage V (2019). Adult spinal deformity. Lancet.

[8] York PJ, Kim HJ (2017). Degenerative Scoliosis. Curr Rev Musculoskelet Med.

[9] Papadopoulos D (2013). Adult scoliosis treatment combining brace and exercises. Scoliosis.

[10] McAviney J, Roberts C, Sullivan B, Alevras AJ, Graham PL, Brown BT (2020). The prevalence of adult de novo scoliosis: a systematic review and meta-analysis. Eur Spine J.

[11] Théroux J, Grimard G, Beauséjour M, Labelle H, Feldman DE (2013). Knowledge and management of adolescent idiopathic scoliosis among family physicians, pediatricians, chiropractors and physiotherapists in Québec, Canada: an exploratory study. J Can Chiropr Assoc.

[12] Eldridge SM, Lancaster GA, Campbell MJ, Thabane L, Hopewell S, Coleman CL, Bond CM (2016). Defining feasibility and pilot studies in preparation for randomised controlled trials: development of a conceptual framework. PLoS One.

[13] Gedda M (2015). French translation of the COREQ reporting guidelines for writing and reading for reporting qualitative research. Kinésithérapie, la Revue.

[14] Tong A, Sainsbury P, Craig J (2007). Consolidated criteria for reporting qualitative research (COREQ): a 32-item checklist for interviews and focus groups. Int J Qual Health Care.

[15] Chiarotto A, Maxwell LJ, Ostelo RW, Boers M, Tugwell P, Terwee CB (2019). Measurement properties of visual analogue scale, numeric rating scale, and pain severity subscale of the brief pain inventory in patients with low Back pain: a systematic review. J Pain.

[16] Fairbank JC, Couper J, Davies JB, O'Brien JP (1980). The Oswestry low back pain disability questionnaire. Physiotherapy.

[17] Vogler D, Paillex R, Norberg M, de Goumoëns P, Cabri J (2008). Cross-cultural validation of the Oswestry disability index in French. Ann Readapt Med Phys.

[18] Alsaleh M, Videloup L, Lobbedez T, Lebreuilly J, Morello R, Thuillier Lecouf A (2019). Improved detection and evaluation of depression in patients with chronic kidney disease: validity and reliability of screening (PHQ-2) and diagnostic (BDI-FS-Fr) tests of depression in chronic kidney disease. Kidney Dis (Basel).

[19] Kroenke K, Spitzer RL, Williams JB (2003). The patient health Questionnaire-2: validity of a two-item depression screener. Med Care.

[20] Demers L, Weiss-Lambrou R, Ska B (1996). development of the Quebec user evaluation of satisfaction with assistive technology (QUEST). Assist Technol.

[21] Demers L, Monette M, Lapierre Y, Arnold DL, Wolfson C (2002). Reliability, validity, and applicability of the Quebec user evaluation of satisfaction with assistive technology (QUEST 2.0) for adults with multiple sclerosis. Disabil Rehabil.

[22] Ostelo RW, de Vet HC (2005). Clinically important outcomes in low back pain. Best Pract Res Clin Rheumatol.

[23] Zaina F, Poggio M, Donzelli S, Negrini S (2018). Can bracing help adults with chronic back pain and scoliosis? Short-term results from a pilot study. Prosthetics Orthot Int.

[24] Weiss HR, Werkmann M (2009). Treatment of chronic low back pain in patients with spinal deformities using a sagittal re-alignment brace. Scoliosis.

[25] Piantoni L, Tello CA, Remondino RG, Bersusky ES, Menéndez C, Ponce C, Quintana S, Hekier F, Francheri Wilson IA, Galaretto E (2018). Quality of life and patient satisfaction in bracing treatment of adolescent idiopathic scoliosis. Scoliosis Spinal Disord.

[26] Wang H, Tetteroo D, Arts JJC, Markopoulos P, Ito K (2021). Quality of life of adolescent idiopathic scoliosis patients under brace treatment: a brief communication of literature review. Qual Life Res.

[27] Law D, Cheung MC, Yip J, Yick KL, Wong C (2017). Scoliosis brace design: influence of visual aesthetics on user acceptance and compliance. Ergonomics.

