# Supplementary material for: Barriers and facilitators to bracing in adults with painful degenerative scoliosis: a single-centred mixed-method feasibility study
Source: BMC Musculoskelet Disord. 2023 Jan 16;24:32. doi: 10.1186/s12891-022-06111-0 (PMC9841704; doi:10.1186/s12891-022-06111-0)

**Appendix 1. STrengthening the Reporting of OBservational studies in Epidemiology (STROBE) statement.**

|  | **Item N°** | **Recommendations** | **Reported on page number** |
| --- | --- | --- | --- |
| **Title and abstract** | 1 | 1. Indicate the study's design with a commonly used term in the title or the abstract 2. Provide in the abstract an informative and balanced summary of what was done and what was found | 1  1-2 |
| **Introduction** | | | |
| Background/  rationale | 2 | Explain the scientific background and rationale for the investigation being reported | 3 |
| Objectives | 3 | State specific objectives, including any prespecified hypotheses | 3 |
| **Methods** | | | |
| Study design | 4 | Present key elements of study design early in the paper | 3-4 |
| Setting | 5 | Describe the setting, locations, and relevant dates, including periods of recruitment, exposure, follow-up, and data collection | 4 |
| Participants | 6 | Give the eligibility criteria, and the sources and methods of selection of participants | 4 |
| Variables | 7 | Clearly define all outcomes, exposures, predictors, potential confounders, and effect modifiers. Give diagnostic criteria, if applicable | 4-5 |
| Data sources/ measurement | 8 | For each variable of interest, give sources of data and details of methods of assessment (measurement). Describe comparability of assessment methods if there is more than one group | 4-5 |
| Bias | 9 | Describe any efforts to address potential sources of bias | 4 |
| Study size | 10 | Explain how the study size was arrived at | 5 |
| Quantitative variables | 11 | Explain how quantitative variables were handled in the analyses. If applicable, describe which groupings were chosen and why | 5 |
| Statistical methods |  | (*a*) Describe all statistical methods, including those used to control for confounding | 4 |
|  | 12 | (*b*) Describe any methods used to examine subgroups and interactions | 4 |
|  |  | (*c*) Explain how missing data were addressed | NA |
|  |  | (*d*) If applicable, describe analytical methods taking account of sampling strategy | NA |
|  |  | (*e*) Describe any sensitivity analyses | NA |
| **Results** | | | |
| Participants |  | (a) Report numbers of individuals at each stage of study-eg numbers potentially eligible, examined for eligibility, confirmed eligible, included in the study, completing follow-up, and analysed | 4 |
|  | 13* | (b) Give reasons for non-participation at each stage | 4 |
|  |  | (c) Consider use of a flow diagram | Fig 1 |
| Descriptive data | 14* | (a) Give characteristics of study participants (eg demographic, clinical, social) and information on exposures and potential confounders | Table 1 |
|  |  | (b) Indicate number of participants with missing data for each variable of interest | NA |
| Outcome data | 15* | Report numbers of outcome events or summary measures | 4 |
| Main results |  | (*a*) Give unadjusted estimates and, if applicable, confounder-adjusted estimates and their precision (eg, 95% confidence interval). Make clear which confounders were adjusted for and why they were included | NA |
|  | 16 | (*b*) Report category boundaries when continuous variables were categorized | NA |
|  |  | (*c*) If relevant, consider translating estimates of relative risk into absolute risk for a meaningful time period | NA |
| Other analyses | 17 | Report other analyses done—eg analyses of subgroups and interactions, and sensitivity analyses | 6 |
| **Discussion** | | | |
| Key results | 18 | Summarise key results with reference to study objectives | 6-7 |
| Limitations | 19 | Discuss limitations of the study, taking into account sources of potential bias or imprecision. Discuss both direction and magnitude of any potential bias | 7 |
| Interpretation | 20 | Give a cautious overall interpretation of results considering objectives, limitations, multiplicity of analyses, results from similar studies, and other relevant evidence | 7 |
| Generalisability | 21 | Discuss the generalisability (external validity) of the study results | 7 |
| **Other information** | | | |
| Funding | 22 | Give the source of funding and the role of the funders for the present study and, if applicable, for the original study on which the present article is based | NA |

**Appendix 2. Consolidated criteria for reporting qualitative research (COREQ) checklist.**

| **COREQ checklist** | | |
| --- | --- | --- |
| **Item number** | **Guide questions/description** | **Pages** |
| **Domain 1: Research team and reﬂexivity** | | |
| *Personal Characteristics* | | |
| 1. Interviewer/facilitator | Which author/s conducted the interview or focus group? | 4 |
| 2. Credentials | What were the researcher’s credentials? E.g. PhD, MD | 1 |
| 3. Occupation | What was their occupation at the time of the study? | 4 |
| 4. Gender | Was the researcher male or female? | 4 |
| 5. Experience and training | What experience or training did the researcher have? | 4 |
| *Relationship with participants* | | |
| 6. Relationship established | Was a relationship established prior to study commencement? | 4 |
| 7. Participant knowledge of the interviewer | What did the participants know about the researcher? e.g. personal goals, reasons for doing the research | 3 |
| 8. Interviewer characteristics | What characteristics were reported about the inter viewer/facilitator? e.g. Bias, assumptions, reasons and interests in the research topic | 3 |
| **Domain 2: study design** | | |
| *Theoretical framework* | | |
| 9. Methodological orientation and Theory | What methodological orientation was stated to underpin the study? e.g. grounded theory, discourse analysis, ethnography, phenomenology, content analysis | 4 |
| *Participant selection* | | |
| 10. Sampling | How were participants selected? e.g. purposive, convenience, consecutive, snowball | 3 |
| 11. Method of approach | How were participants approached? e.g. face-to-face, telephone, mail, email | 4 |
| 12. Sample size | How many participants were in the study? | 5 |
| 13. Non-participation | How many people refused to participate or dropped out? Reasons? | 5 |
| *Setting* | | |
| 14. Setting of data collection | Where was the data collected? e.g. home, clinic, workplace | 5 |
| 15. Presence of non-participants | Was anyone else present besides the participants and researchers? | 4 |
| 16. Description of sample | What are the important characteristics of the sample? e.g. demographic data, date | Table 1 |
| *Data collection* | | |
| 17. Interview guide | Were questions, prompts, guides provided by the authors? Was it pilot tested? | 4  Appendix 2 |
| 18. Repeat interviews | Were repeat inter views carried out? If yes, how many? | 4 |
| 19. Audio/visual recording | Did the research use audio or visual recording to collect the data? | 4 |
| 20. Field notes | Were ﬁeld notes made during and/or after the interview or focus group? | 4 |
| 21. Duration | What was the duration of the inter views or focus group? | 4 |
| 22. Data saturation | Was data saturation discussed? | 7 |
| 23. Transcripts returned | Were transcripts returned to participants for comment and/or correction? | 4 |
| **Domain 3: analysis and ﬁndings** | | |
| *Data analysis* | | |
| 24. Number of data coders | How many data coders coded the data? | 4 |
| 25. Description of the coding tree | Did authors provide a description of the coding tree? | Tables 2 and 3 |
| 26. Derivation of themes | Were themes identiﬁed in advance or derived from the data? | 4 |
| 27. Software | What software, if applicable, was used to manage the data? | NA |
| 28. Participant checking | Did participants provide feedback on the ﬁndings? | 4 |
| *Reporting* |  |  |
| 29. Quotations presented | Were participant quotations presented to illustrate the themes/ﬁndings? Was each quotation identiﬁed? e.g. participant number | Tables 2 and 3 |
| 30. Data and ﬁndings consistent | Was there consistency between the data presented and the ﬁndings? | 5 |
| 31. Clarity of major themes | Were major themes clearly presented in the ﬁndings? | Tables 2 and 3 |
| 32. Clarity of minor themes | Is there a description of diverse cases or discussion of minor themes? | Tables 2 and 3 |

**Appendix 3. Checklist for Reporting Results of Internet E-Surveys (CHERRIES).**

| **Item Category** | **Checklist Item** | **Explanation** |
| --- | --- | --- |
| Design | Describe survey design | It was a cross-sectional survey. The target population was people older than 40 years old with degenerative or de novo scoliosis followed at Cochin Hospital. |
| Institutional Review Board (IRB) approval and informed consent process | IRB approval | NA |
|  | Informed consent | All participants were informed orally or in writing by the investigator of the design and purpose of the study |
|  | Data protection | Patient demographic analyzes were performed at the hospital after registration with the General Data Protection Regulation |
| Development and pre-testing | Development and testing | Questionnaires were elaborated by 2 physicians specialized in physical and rehabilitation medicine (RPM) and tested on two patients |
| Recruitment process and description of the sample having access to the questionnaire | Open survey vs closed survey | Closed survey, a secured link was sent to each participant |
|  | Contact mode | Initial contact was made during equipment consultation |
|  | Advertising the survey | No advertising was made. |
| Survey administration | Web/E-mail | The questionnaires were stored on a website, with automatic method for capturing responses in the database. |
|  | Context | Only participants contacted by mail received the link to the secured online platform. |
|  | Mandatory/voluntary | It was a voluntary survey. |
|  | Incentives | No incentives were used. |
|  | Time/Date | Between July 2020 and September 2020 |
|  | Randomization of items or questionnaires | No randomization planned or needed for the purpose of this study |
|  | Adaptive questioning | No randomization planned or needed for the purpose of this study |
|  | Number of items | Pre final questionnaire included: 29 items   - Digital lumbar pain scale 1 - Digital radicular pain scale 1 - Oswestry disability index 10 - PHQ2 scale 2 - QUEST questionnaire 12 - Additional questions for patients test 3   Final questionnaire was the same as the pre final |
|  | Number of screens (pages) | 5 pages |
|  | Completeness check | For the pre final and final questionnaires, completeness was checked before the questionnaire has been submitted, all the items were mandatory |
|  | Review step | Patients did not have the possibility to have several accesses to complete or modify their answers. |
| Response rates | Unique site visitor | Each participant had a unique access-link. Patient’s answers were saved under their anonymization number. |
|  | View rate (ratio unique site visitors/unique survey visitors) | NA. Only patients of the survey could access to the internet platform. |
|  | Participation rate (ratio unique survey page visitors/agreed to participate) | For the pre-final questionnaires: 2/3 invited participants answered (67% of answer rate)  For the final-test: 14/24 (58%) invited participants answered |
|  | Completion rate (ratio agreed to participate/finished survey) | For the pre-final and final questionnaire the completion rate was 100% |
| Preventing multiple entries from the same individual | Cookies used | No cookies were used. |
|  | IP check | IP addresses were not checked. |
|  | Log file analysis | NA |
|  | Registration | Patient's answers were registered with an anonymization number corresponding to their order to response. |
| Analysis | Handling of incomplete questionnaires | All questionnaires were analyzed |
|  | Questionnaires submitted with atypical timestamp | The time needed to fill in a questionnaire was not used to exclude questionnaires |
|  | Statistical correction | NA |

**Appendix 4. Interview guide.**

**Presentation**

Hello,

Dr Marie-Ombeline Chagnas, I am a physician in the Physical Medicine and Rehabilitation Department of Cochin Hospital. Following your fitting consultation, I am carrying out a study with patients to better understand the elements that can be obstacles to wearing a corset and the solutions that can be provided.

Would you be willing to answer a few questions now? Or would you like to schedule a telephone interview on a day that is more convenient for you?

The data collected during our interview will be anonymized and used for research purposes if you agree.

Our conversation will be recorded so that the responses can be properly transcribed and analyzed.

**Part 1: Semi-qualitative part**

Method: Telephone interview with Dr Marie-Ombeline Chagnas

**1. Making the fitting:**

You were seen in a fitting consultation at the Cochin hospital on … (put the date)

***What type of brace do you wear?***

***- On what date did you receive the final brace?***

***- If you refused to have a brace made: for what reason?***

**2. Acceptability of wearing a brace:**

***1) Do you wear your brace as often as recommended?***

***2) How many days per week do you wear your brace on average?***

***3) On average, how many hours per day do you wear this brace?***

***4) Under what circumstances or activities do you wear it?***

***5) Have you integrated your brace into your daily life?***

***6) Do you ever forget to put on your brace?***

***7) Do you ever forget that you are wearing a brace?***

3. **Side effects and self-reported solutions provided by the patient himself:**

Did wearing the brace cause any adverse effects?

**Open-ended question, then complete the checklist:**

|  | Yes | No |
| --- | --- | --- |
| ***Pain caused by the brace? (location of the pain)*** |  |  |
| ***Skin lesions?*** |  |  |
| ***Discomfort due to temperature changes (e.g. sweating in summer)?*** |  |  |
| ***Respiratory discomfort when wearing the brace?*** |  |  |
| ***Digestive discomfort with the brace?*** |  |  |
| ***Difficulty in finding clothes adapted to wearing the brace?*** |  |  |
| ***Appearance of urinary urgency?*** |  |  |
| ***A feeling of claustrophobia?*** |  |  |
| ***Other undesirable effects*** |  |  |

***Was the brace readjusted by the ortho-prosthesist?***

**4. Obstacles and solutions proposed by the patient himself :**

Have you encountered any obstacles to wearing your brace?

**Open-ended question then complete with the checklist:**

|  | Yes | No |
| --- | --- | --- |
| ***Pain related to the spinal deformity? (location of the pain)*** |  |  |
| ***A limitation in the activities of daily living: which ones?*** |  |  |
| ***The aesthetic aspect of the brace: specify?*** |  |  |
| ***The weight of the brace*** |  |  |
| ***The cleaning of the brace*** |  |  |
| ***Difficulty putting on and/or removing the brace: what was the origin of this difficulty?*** |  |  |
| ***Difficulty to sit down, to keep a prolonged sitting position, to drive?*** |  |  |
| ***The presence of an inguinal or umbilical hernia?*** |  |  |
| ***Other obstacles*** |  |  |

***For each obstacle: how would you improve this limitation to wearing the brace? What solution have you brought yourself?***

**5. Levers for wearing the brace:**

What elements have encouraged you or would encourage you to wear the brace?

|  | Yes | No |
| --- | --- | --- |
| ***An effective relief of lumbar and/or radicular pain.*** |  |  |
| ***A decrease in dyspnea related to the spinal deformity.*** |  |  |
| ***A straightening of the spine and a horizontal gaze.*** |  |  |
| ***An aesthetic aspect, in connection with the correction brought by the apparatus.*** |  |  |
| ***A facilitation of mobility in the activities of daily life*** |  |  |
| ***A feeling of overall comfort*** |  |  |
| ***An increase in your walking perimeter*** |  |  |
| ***An improvement of your autonomy*** |  |  |
| ***A reduction in fatigue (due to straightening efforts)*** |  |  |
| ***A satisfaction of the people around you.*** |  |  |

Appendix 5. Disability according to Oswestry disability index (n=13).


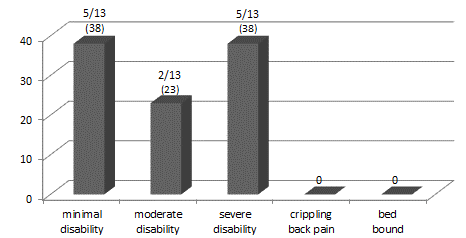


Appendix 6. Symptoms of depression according to the patient health questionnaire-2 (n=13).

| Score < 3, n (%) | 8 (61) |
| --- | --- |
| Score ≥ 3, n (%) | 5 (38) |

**Appendix 7. Satisfaction with bracing according to the Quebec user evaluation of satisfaction with assistive technology (n=13).**


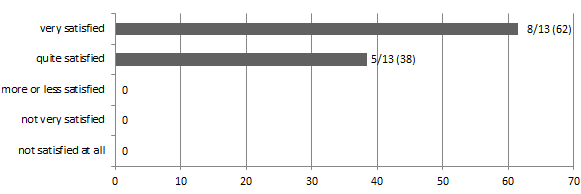

Supplement: Supplementary file 1 — Additional file 1: Appendix 1. Strengthening the Reporting of OBservational studies in Epidemiology (STROBE) statement. Appendix 2. Consolidated criteria for reporting qualitative research (COREQ) checklist. Appendix 3. Checklist for Reporting Results of Internet E-Surveys (CHERRIES). Appendix 4. Interview guide. Appendix 5. Disability according to Oswestry disability index (n = 13). [file 12891_2022_6111_MOESM1_ESM.docx]
